# Untangling the biological effects of cerium oxide nanoparticles: the role of surface valence states

**DOI:** 10.1038/srep15613

**Published:** 2015-10-22

**Authors:** Gerardo Pulido-Reyes, Ismael Rodea-Palomares, Soumen Das, Tamil Selvan Sakthivel, Francisco Leganes, Roberto Rosal, Sudipta Seal, Francisca Fernández-Piñas

**Affiliations:** 1 Departamento de Biología, Facultad de Ciencias, Universidad Autónoma de Madrid, E-28049, Spain; 2 Advanced Material Processing Analysis Center and Nanoscience Technology Center, Materials Science and Eng, UCF College of Medicine, University of Central Florida, Florida 32826, United States; 3 Departamento de Ingeniería Química, Universidad de Alcalá, E-28871, Alcalá de Henares, Madrid, Spain

## Abstract

Cerium oxide nanoparticles (nanoceria; CNPs) have been found to have both pro-oxidant and anti-oxidant effects on different cell systems or organisms. In order to untangle the mechanisms which underlie the biological activity of nanoceria, we have studied the effect of five different CNPs on a model relevant aquatic microorganism. Neither shape, concentration, synthesis method, surface charge (ζ-potential), nor nominal size had any influence in the observed biological activity. The main driver of toxicity was found to be the percentage of surface content of Ce^3+^ sites: CNP1 (58%) and CNP5 (40%) were found to be toxic whereas CNP2 (28%), CNP3 (36%) and CNP4 (26%) were found to be non-toxic. The colloidal stability and redox chemistry of the most and least toxic CNPs, CNP1 and CNP2, respectively, were modified by incubation with iron and phosphate buffers. Blocking surface Ce^3+^ sites of the most toxic CNP, CNP1, with phosphate treatment reverted toxicity and stimulated growth. Colloidal destabilization with Fe treatment only increased toxicity of CNP1. The results of this study are relevant in the understanding of the main drivers of biological activity of nanoceria and to define global descriptors of engineered nanoparticles (ENPs) bioactivity which may be useful in safer-by-design strategies of nanomaterials.

Cerium (Ce) is a rare-earth element which belongs to the lanthanide series. In solution Ce may exist as Ce^3+^ and Ce^4+^ and depending on environmental conditions can cycle between both oxidation states^1^. Ce may also exist as oxide form, CeO_2_, which has been used widely as glass polishing^2^ and is used as fertilizer in Chinese agriculture for crop improvement^3^. As a further advance in Ce applications, nanoceria or cerium oxide nanoparticles (CNPs) are gaining interest in many industrial fields^4^,^5^,^6^, in plants to increase photosynthesis *via* suppression of reactive oxygen species (ROS)^7^; also as an antioxidant, there are a number of reports in biomedicine concerning the protective effects of nanoceria in certain neurological disorders^8^; to provide cells protection from radiation^9^ and to be cytotoxic to cancer cells^10^.

Although nanoceria shares similar physicochemical properties with bulk cerium, nanoceria has a large number of point surface defects^11^ which correspond mainly to oxygen vacancies at the surface of the nanoparticle lattice^12^. These defects explain the autocatalytic properties of nanoceria, Esch *et al.*^13^ determined that Ce^3+^ atoms occupy the center of the oxygen vacancies surrounded by Ce^4+^ atoms. This particular configuration seems to underlie the unique redox-chemistry of nanoceria which is able to switch oxidation states between Ce^3+^ and Ce^4+^ depending on the prevailing environmental conditions^14^. This reduction/oxidation behavior is responsible for the observed antioxidant properties of nanoceria. It has been found that nanoceria displays superoxide-dismutase mimetic activity^15^, catalase mimetic activity^16^ or the capacity to scavenge nitric oxide radicals^17^. These antioxidant activities have been shown to depend on the Ce^3+^/Ce^4+^ ratio at the particle surface. In this regard, it has been reported that exposure of CNPs to phosphate shifted the redox state and altered their catalytic properties *in vitro*^18^,^19^.

On the other side of the coin, nanoceria has also been found to display oxidase activities^20^ and to generate damaging oxygen radicals in a range of organisms and cell systems^14^,^21^,^22^,^23^,^24^,^25^,^26^.

Besides cerium valence states at the surfaces of CNPs, there are a number of factors which may influence CNPs interaction and thus biological effects on living cells. In this regard, a deep understanding of the colloidal chemistry of nanoceria (ζ-potential, solution pH, use of dispersants, particle size, *etc*.) in the tested biological media is of outmost relevance, processes such as aggregation/agglomeration have been found to modulate the toxicity of CNPs in aquatic organisms^24^,^25^,^27^,^28^,^29^. Another relevant factor could be the dissolution of free Ce^3+^ in CNPs suspensions, although most studies have found that this phenomenon in most biological media is almost negligible and given the low intrinsic toxicity of Ce^3+^ does not account for the observed toxicity of nanoceria^24^,^25^,^27^,^29^. CNPs internalization has been found to be an issue mostly in human cell lines^10^, however, in environmentally relevant organisms such as microalgae, no cell internalization has been reported^25^,^28^,^29^.

As there are so many contradicting reports regarding the effects of nanoceria and so many factors which may contribute to the biological effects, the aim of this study was to perform a thorough study of the effect of five different CNPs in an effort to untangle the mechanisms which underlie the biological activity of nanoceria. The study was performed on a model aquatic microorganism, the green alga *Pseudokirchneriella subcapitata*.

The tested nanoceria have defined percentages of surface content of Ce^3+^ sites, they show distinct morphologies (spheres, rods or cubes) and different nominal sizes. In addition, they were synthesized by two different methods. Furthermore, to get deeper insights into the biological mechanisms of CNPs, the colloidal stability and redox chemistry of the most and least toxic CNPs were modified and the effect of these modifications on the model organism were tested.

## Results

### Physicochemical characterization of CNPs

**Figure 1:**
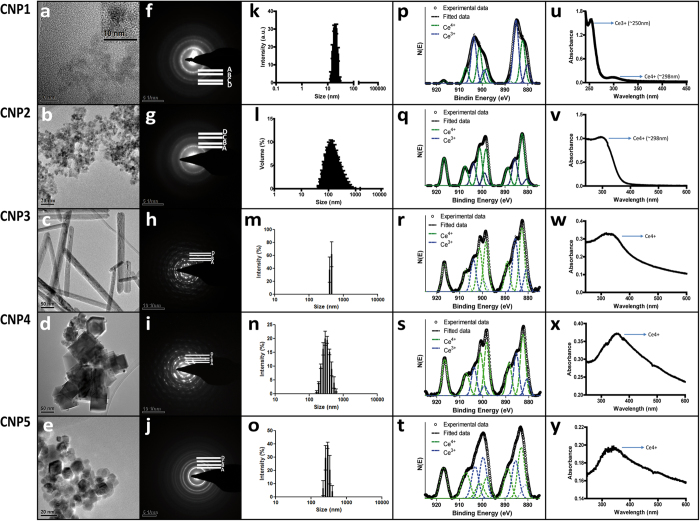
Characterization of synthesized CNPs. (**a**–**e**) photographs show TEM images of the five CNPs (inset on CNP1: high magnification image). (**f–j**) Show selected areas of electron diffraction pattern of CNPs (different crystal planes of fluorite crystal structure). (**k–o**) Graphs represent the nanoparticle hydrodynamic radius. (**p–t**) Images show deconvoluted XPS spectra of all CNPs. The UV−vis absorption spectra of CNPs suspension is represented in (**u–y**).

The different CNPs were synthesized by two different methods and with varying % surface Ce^3+^, morphology and sizes. Figure 1a–e show particle sizes and shapes of all CNPs as observed by Transmission Electron Microscopy (TEM). CNP1, CNP2 and CNP5 had spheres shape with diameters approximately of 5, 7 and 18 nm, respectively. CNP3 had rod shape (350 nm long with a width of 20 nm) and CNP4 had cubic shape with a particle size around 50 nm. Figure 1f–j show selected area electron diffraction (SAED) patterns which confirm the crystalline nature and fluorite structure of the nanoparticles. A(111), B(200), C(220) and D(311) in SAED correspond to the different lattice planes of fluorite crystal structure. Figure 1 k–o show the hydrodynamic sizes of all CNPs in ddH_2_O. As can be seen in the figure, hydrodynamic size ranged from 20 to 350 nm, depending on morphology. The deconvoluted Ce (3d) XPS spectra of all CNPs are also shown in Fig. 1 p–t. CNP1 had the highest % surface Ce^3+^ (58%) followed by CNP5 (40%), CNP3 (36%), CNP2 (28%) and CNP4 (26%). Figure 1 u–y illustrate UV-visible absorbance spectra of CNPs. Absorbance spectra fluctuated depending on the % surface content of Ce^3+^ and Ce^4+^ of each nanoparticle^30^. A strong absorption at 250 nm was observed in CNP1, which is directly related to Ce^3+^^31^. The other CNPs (CNP2-5) had a maximum of absorption at 298 nm, indicative of their higher Ce^4+^ content.

Table 1 shows the physicochemical characteristics of the five CNPs at 10 mg/L suspended in either ddH_2_O or OECD algal exposure medium. Measured ζ-potential values of CNP1, CNP2, CNP3, and CNP5 in ddH_2_O were positive with the exception of CNP4 which showed a high negative value. However, when the CNPs were suspended in OECD algal medium, there was a shift to negative values for CNP1, CNP2, CNP3, and CNP5. CNP1 (−12.2 mV) had the lowest absolute values of all samples (near to neutrality) indicating a less stable suspension as compared to the other tested CNPs. The other CNPs had more negative values, around −22 mV which indicated that they were stably dispersed^32^,^33^.

Furthermore, it is worth noticing that CNPs appeared aggregated in OECD medium with higher sizes than in ddH_2_O, a phenomenon frequently found in the literature due to the complexity and usually high conductivity of most culture media^25^,^28^,^29^. According to DLS measurements, the effective diameters were between 250–795 nm (see Table 1). CNP1 had the largest effective diameter indicating increased agglomeration/aggregation under these conditions which is in agreement with a less stable suspension as stated above.

Spontaneous cerium dissolution in the exposure media was tested for all CNPs by performing ICP-MS analyses of ultrafiltrated samples. The results indicated negligible (<0.0008 mg/L for 10 mg/L of each tested CNP; Supplementary Table S1) amounts of dissolved Ce in OECD medium.

### Toxicity of the CNPs towards *P. subcapitata*

**Figure 2:**
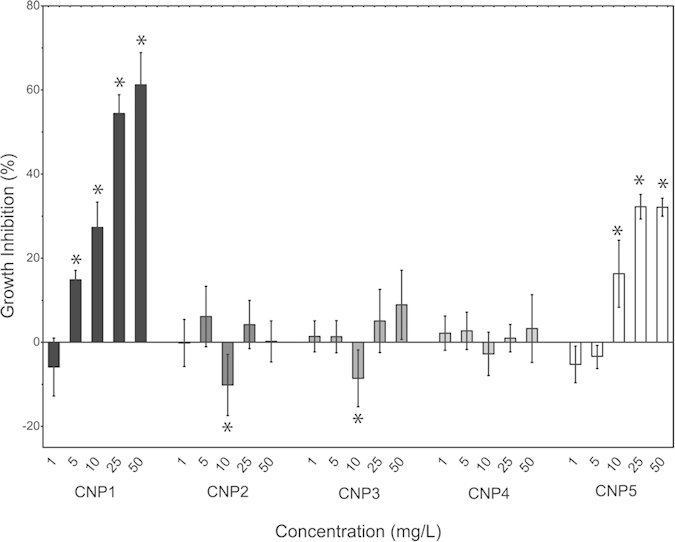
Effect of 72 h exposure to the five CNPs on growth of *P. subcapitata.* Data are expressed as percentages of the value of untreated cells (mean ± standard deviation). Statistically significant differences (*p* *<* *0.05*) are marked by asterisks.

The effect of the CNPs on growth of *P. subcapitata* was assessed by measuring *in-vivo* fluorescence of chlorophyll^32^. Results of exposure to the five CNPs in the concentration range of 1 to 50 mg/L are shown in Fig. 2. Growth inhibition significantly (*p* *<* *0.05*) increased with concentrations higher than 1 mg/L for CNP1 and 5 mg/L for CNP5. But, while the highest algal growth inhibition produced by CNP5 was approximately 30% at 50 mg/L, CNP1 reached a maximum growth inhibition of near 65% at the same concentration. In contrast, the growth of *P. subcapitata* was not affected significantly by CNP2, CNP3 and CNP4 exposure, even at the highest concentration tested (50 mg/L). Interestingly, CNP2 and CNP3 statistically (*p* *<* *0.05*) stimulated the growth of *P. subcapitata* at a concentration of 10 mg/L. In conclusion, from the five tested CNPs, only CNP1 and CNP5 caused algal growth inhibition, with CNP1 as the most toxic cerium oxide nanoparticle. For none of these CNPs, dissolved free Ce^3+^ explained the observed toxicity as the concentrations found by ICP-MS did not exert any toxic effect (Supplementary Figure S1).

**Figure 3:**
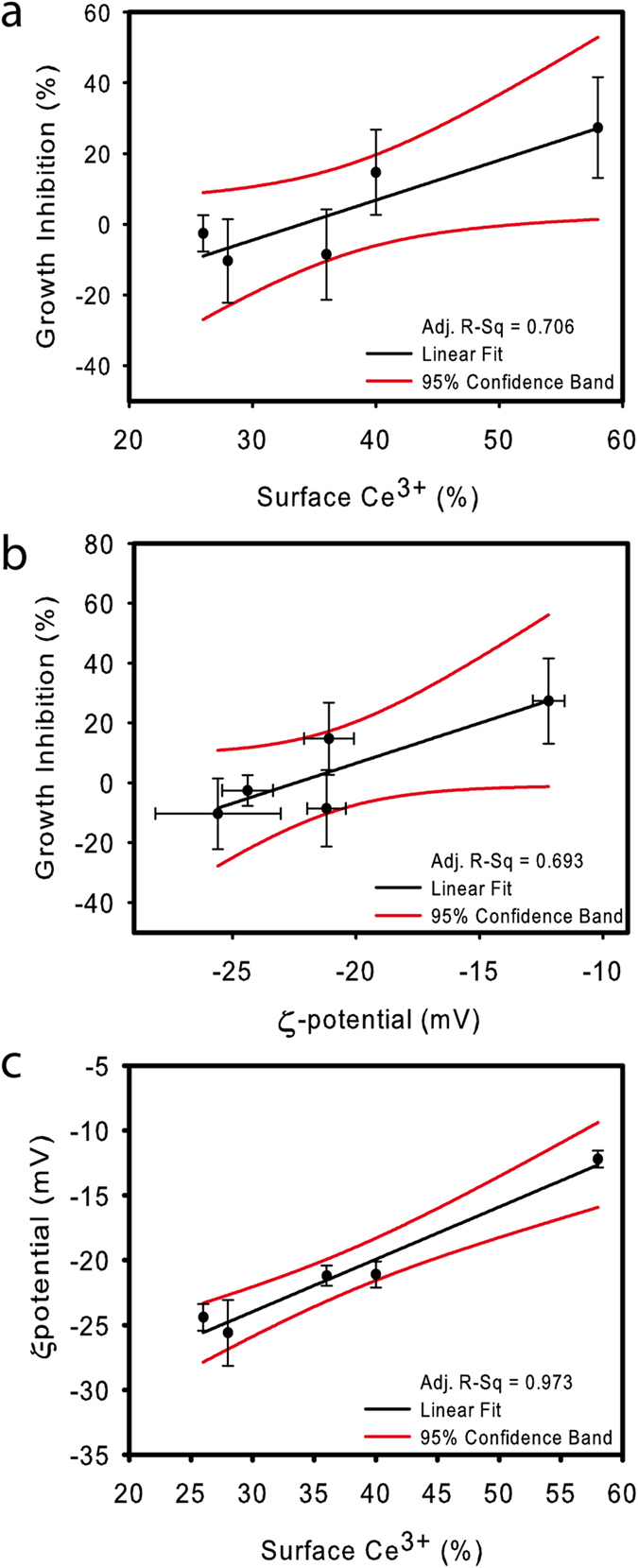
Correlation analyses. (**a**) Correlation between algal growth inhibition (%) caused by CNPs exposure and % surface Ce^3+^ of each tested CNP. (**b**) Correlation between algal growth inhibition (%) and ζ-potential (mV) in OECD-medium. (**c**) correlation between ζ-potential (mV) in OECD-medium and % surface Ce^3+^ of each tested CNP.

From the data in Table 1 and those in Fig. 2, it appears that some particle properties, such as % surface Ce^3+^, ζ-potential or effective diameter may probably correlate with toxicity, but other parameters such as nominal size or shape might also be involved. Thus, correlation analyses were performed in order to identify which physicochemical property of the tested CNPs, if any, might explain the observed biological effects. Nominal size, effective diameter, nanoparticle ζ-potential, shape and % surface Ce^3+^ were evaluated as potential explanatory variables (Fig. 3, Supplementary Table S2 and Supplementary Figure S2). Both % surface Ce^3+^ and ζ-potential correlated equally well (*R*^*2*^ *≈* *0.7* with significant *p*-values) with algal growth inhibition (Fig. 3a,b; Supplementary Table S2). Size did not show any significant correlation (Supplementary Table S2) and regarding shape, as it is a categorical variable, a different statistical approach was followed. Algal growth was only significantly (ANOVA, α = 0.05) affected by spherical nanoceria: CNP1, CNP2 and CNP5 (Supplementary Figure S2a) and as also shown in Supplementary Figure S2b, within spheres, CNP1 and CNP5 with the higher % surface Ce^3+^ significantly inhibited growth, while CNP2 with a lower % surface Ce^3+^ slightly stimulated growth (already shown in Fig. 2). So, shape *per se* did not affect toxicity; in fact, CNP2, CNP3 and CNP4 which were non-toxic had different morphology: sphere, rod and cube, respectively.

Both parameters % surface Ce^3+^ and ζ-potential measured in OECD medium presented a very high and statistically significant correlation (*R*^2^ *≈* *0.97, p* *=* *0.001*) (Fig. 3C). However, to our knowledge there is no previous evidence of such a direct influence of surface Ce^3+^ on ζ-potential of cerium oxide nanoparticles. It may be possible that % surface Ce^3+^ may alter the state of nanoceria in a specific medium and thus, may affect toxicity. In this regard, it is well known that surface chemistry of nanoparticles can alter their physicochemical properties in fluids, such as ζ-potential and colloidal stability^34^ and it is important to note that measured ζ-potential values are quite different in OECD algal medium as compared to distilled water (See Table 1). Therefore, either ζ-potential or % surface Ce^3+^ might explain the observed toxicity pattern. However, which is the actual driving factor of the observed toxicity? Are the differential toxic responses driven by the distinct % of surface Ce^3+^ and Ce^4+^ content? Or, are the observed differences driven by the found divergences in ζ-potential which usually correlate with the colloidal stability of the CNPs suspensions^32^? Are both factors responsible?

### Untangling the main drivers of CNP bioactivity

In order to answer those questions, CNP1 and CNP2 were chosen to further understand which parameter(s) caused the observed toxicity. Both CNPs were synthesized by the same method and had similar sizes and shapes but clearly differed in their biological effect (CNP1 as the most toxic and CNP2 as non-toxic) and in the above identified physicochemical properties of interest (ζ-potential and % surface Ce^3+^).

Previous works^32^,^34^,^35^ have shown that the addition of specific chemical agents influence nanoparticle surface charge and may have significant effects on colloidal stability and surface chemistry^18^,^36^. Stock suspensions of the CNP1 and CNP2 were prepared with specific modifying chemical agents in order to provoke specific changes in colloidal stability and surface reactivity. ζ-potential was modified by using trivalent iron (Fe^3+^) which is specifically adsorbed on negatively charge surfaces and can neutralize the negative surface charges altering the tendency to homo and/or heteroaggregation of nanoparticles^32^,^34^. The intrinsic reactivity of Ce^3+^, generated by its surface catalytic properties, was masked by using phosphate ions (PO_4_^3−^)^18^. PO_4_^3−^ was used to block the redox cycling between Ce^3+^ and Ce^4+^ at the particle surface due to strong association among surface Ce^3+^ with phosphate anions^18^,^37^.

In addition to growth inhibition studies, additional biological characterizations were performed to get a deeper insight into the toxicological mechanisms involved. For this, nano-bio interactions were tracked by flow cytometry, TEM-XEDS and FTIR analysis. As oxidative stress has been found to be a basic mechanism of CNP toxicity^21^,^23^,^26^, intracellular ROS production was also evaluated.

As shown in Supplementary Table S3, Fe treatment induced charge reversal on CNP1 (from −12.2 mV to 10.15 mV) and a reduction of 10 mV (in absolute values) on CNP2 surface charge (from −25.6 mV to −15.3 mV) (See Table 1 for comparison). Besides, Fe treatment significantly increased the effective diameter for CNP2, but did not increase the effective diameter of CNP1 which was already high, although Fe increased its PDI. These changes induced by Fe treatment indicated colloidal destabilization^32^,^34^. The addition of the phosphate buffer did not induce significant changes in the nanoparticle suspensions. According to Singh *et al.*^18^, phosphate shifts the redox state of CNPs but does not significantly alter their colloidal stability.

**Figure 4:**
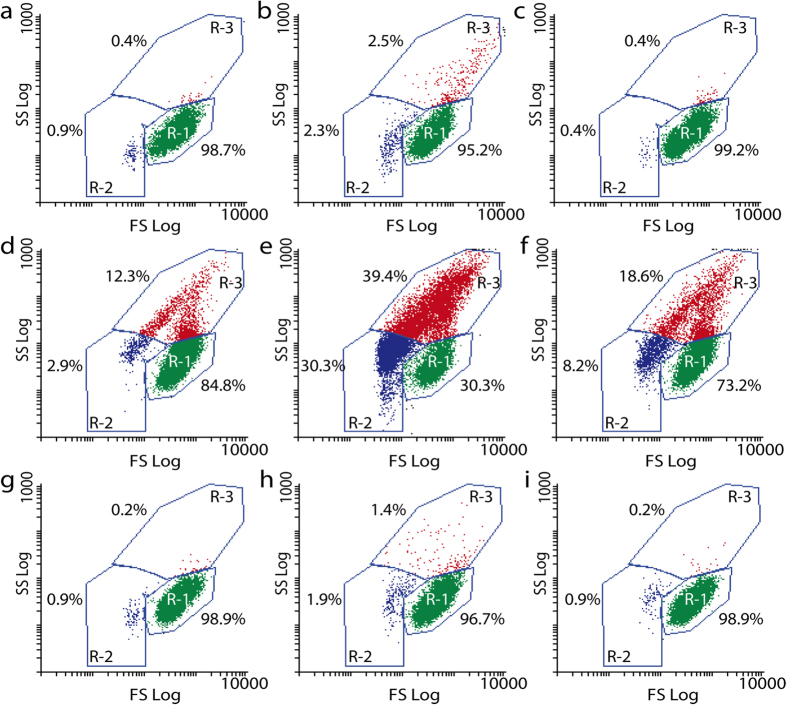
Flow Cytometry plots of cell complexity (SS) as function of cell size (FS) of *P. subcapitata* exposed during 48 h to CNP1 and CNP2 at 10 mgL^−1^. Control cells: (**a**) cells without treatment, (**d**) cells + Fe, (**g**), cells+PO_4_^3−^, cells exposed to CNP1 (**b**), CNP2 (**c**), CNP1+Fe (**e**), CNP2+Fe (**f**), CNP1+PO_4_^3−^ (**h**) and CNP2+PO4^3−^ (**i**).

When cells and nanoparticles co-occur, hetero-aggregation has been frequently observed, in particular in those systems where nanoparticles show some degree of colloidal destabilization^26^,^29^,^32^. Potential hetero-aggregation between algal cells and CNP1/CNP2 was tracked by using Flow Cytometry. Figure 4 shows flow cytograms of cell complexity (internal granularity) as a function of cell size of *P. subcapitata* (control), *P. subcapitata* exposed to CNP1 and CNP2 (Fig. 4b,c) and *P. subcapitata* exposed to both CNPs after the treatment with Fe (Fig. 4e,f) and phosphate (Fig. 4h,i). As can be seen, the flow cytograms of algal cells (control) exhibited a defined ellipsoidal population (denoted as subpopulation R-1) with 98.7% of cells inside this region. CNP1 exposure reduced the percentage of cells inside subpopulation R-1 (95.2% of total cells), showing a shift to the left indicating a subpopulation of cells (denoted as R-2) with lower size and diminished cell size/complexity which might indicate highly damaged cells or cell death. In addition, a clearly significant shift to the upper left (subpopulation denoted as R-3 which reached 2.5% of total cells) was also observed which indicated an increase in cell size and complexity that could be interpreted as the formation of nanoparticle-cell hetero-aggregates^26^,^32^. As expected, CNP2 did not induce hetero-aggregation with algal cells and the flow cytogram was quite similar to that of the non-exposed cells. The treatment with Fe already increased the cell size and complexity of non-exposed cells (subpopulation R-3: 12.3%) indicating that the Fe treatment *per se* induced a slight flocculation of algal cells. It also slightly increased subpopulation R-2 (2.9%), indicating some extent of cell damage. There was a clear and remarkable change in the populations of algal cells exposed to CNP1 and Fe as this treatment drastically shifted the main population to the upper left of the cytogram (subpopulation R-3) with 39.4% of total cells showing higher complexity and size. This is probably due to the formation of numerous nanoparticle-cell hetero-aggregates due to the increased colloidal destabilization of CNP1. Fe treatment also provoked and increase in the R-2 subpopulation (it reached 30.3% of total cells) indicating increased cell damage. CNP2-Fe treatment exhibited smaller changes than CNP1-Fe with respect to the control: R-3 and R-2 slightly increased (reaching 18.6% and 8.2% respectively) indicating that some extent of heteroaggregation and cell damage were occurring for CNP2 treated with Fe. Phosphate treatment did not have any significant effect on cell size or complexity of non-exposed cells or cells exposed to CNP2. However, it significantly decreased the subpopulation of cells with increased cell size and complexity (R-3 comprising only 1.4% of total cells) observed in cells exposed to CNP1 without treatments (Fig. 4b), indicating that phosphate treatment inhibited to some degree the hetero-aggregation process. It also decreased the R-2 subpopulation of damaged cells to 1.9% of total cells, probably indicating a decrease in toxicity.

**Figure 5:**
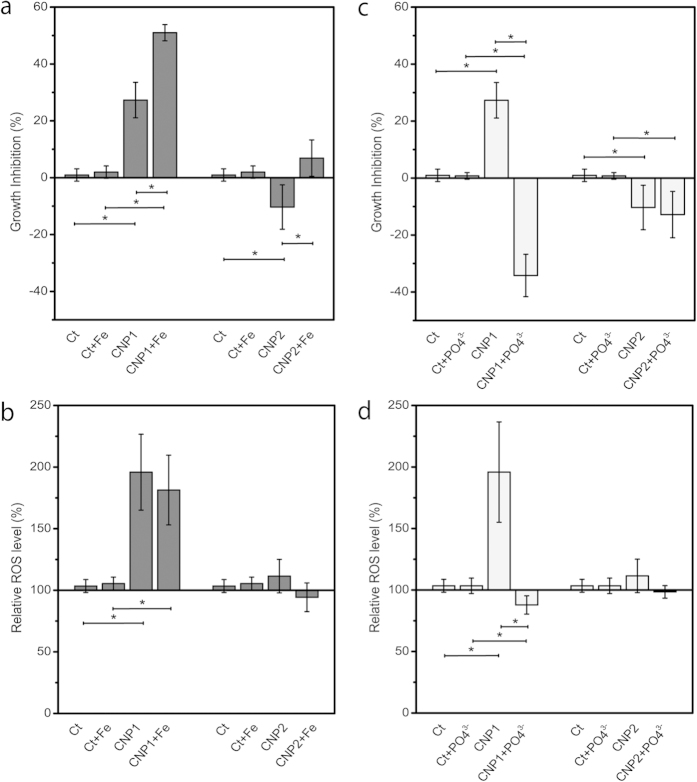
Effect of combined treatments of CNP1 and CNP2 with Fe (left column) or PO_4_^3–^ (right column) on the growth of *P. subcapitata* during 72 h (a,c), and intracellular ROS production (b,d). Mean ± standard deviation. Statistically significant differences (*p* *<* *0.05*) are marked by asterisks.

In order to correlate the observed hetero-aggregation patterns with toxicity, the effect of the combined treatments CNP1-Fe, CNP2-Fe, CNP1-phosphate and CNP2-phosphate on growth and ROS formation in exposed cells was studied. As can be seen in Fig. 5a, Fe treatment significantly (*p* *<* *0.05*) increased growth inhibition in CNP1 exposed cells. In the case of CNP2, Fe treatment caused a slight inhibition of growth as compared to the observed growth stimulation when exposed to CNP2 alone. These results correlated with those of the flow cytograms. CNP1 also caused significant (*p* *<* *0.05*) ROS formation in exposed cells (Fig. 5b), indicating that oxidative stress might be an important mechanism of toxicity. However, Fe treatment did not increase ROS further. CNP2 alone or combined with Fe did not induce ROS formation in the exposed cells. CNP1-Phosphate treatment (Fig. 5c,d) completely reverted the toxicity of CNP1 resulting in a significant (*p* *<* *0.05*) growth stimulation. Moreover, phosphate treatment slightly reduced the ROS levels of the non-exposed cells (Fig. 5d). CNP2 treated with phosphate maintained its non-toxic profile (Fig. 5c,d).

The drastic effect of phosphate treatment on the toxic patterns of cells exposed to CNP1 might be due to the affinity of surface Ce^3+^ for phosphate anions, which occupy surface oxygen vacancies forming CePO_4_; this modifies the nanoceria surface and blocks the redox cycling between Ce^3+^ and Ce^4+^^18^. The fact that phosphate treatment totally reverted the toxicity of CNP1 indicates that the % surface Ce^3+^ is probably the main driver of CNP1 toxicity. This is further demonstrated by the fact that CNP2 with a significantly lower % surface Ce^3+^ was not toxic even under Fe treatment which induced heteroaggregation between cells and nanoparticles.

**Figure 6:**
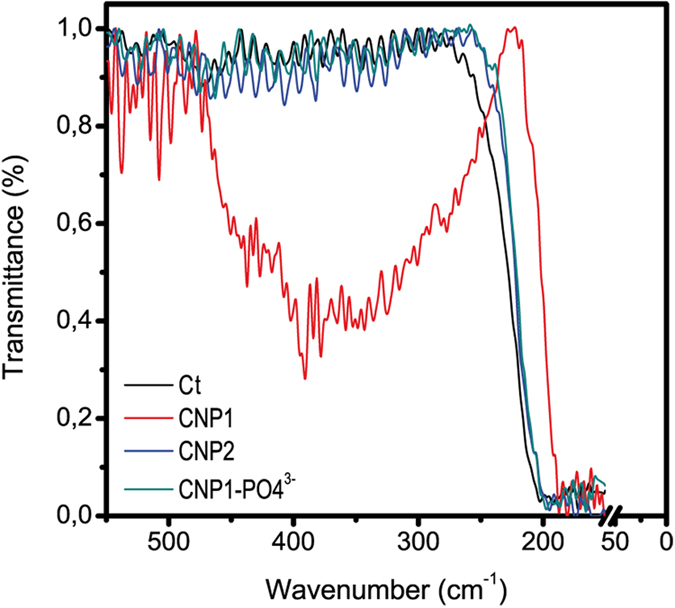
FTIR transmission profiles of *P. subcapitata* control cells (Ct), CNP1, CNP2 and CNP1-PO_4_^3−^. Wavenumber range: 550–50 cm^−1^.

Direct attachment of nanoparticles to cellular envelopes has been found to mediate toxicity of several nanoparticles to algae in which internalization has not been found^25^,^28^,^32^. In order to confirm whether % surface Ce^3+^ might influence CNP attachment to algal cell envelope, FTIR and TEM-XEDS studies were made. As shown by the FTIR profiles in Fig. 6, the presence of CNP1 was detected in the envelope of *P. subcapitata* (absorption band at wavenumber 400 cm^−1^, representing the Ce-O stretch^38^; no significant differences were found at wavenumbers in the range 4000–550 cm^−1^) (not shown). No absorption peaks at wavenumber of 400 cm^−1^ or higher were found for CNP2 or CNP1 treated with phosphate indicating that treatment with phosphate which blocks Ce^3+^, prevents CNP1 attachment to the cell wall. As expected, the non-toxic CNP2 did not attach to the cell envelope.

**Figure 7:**
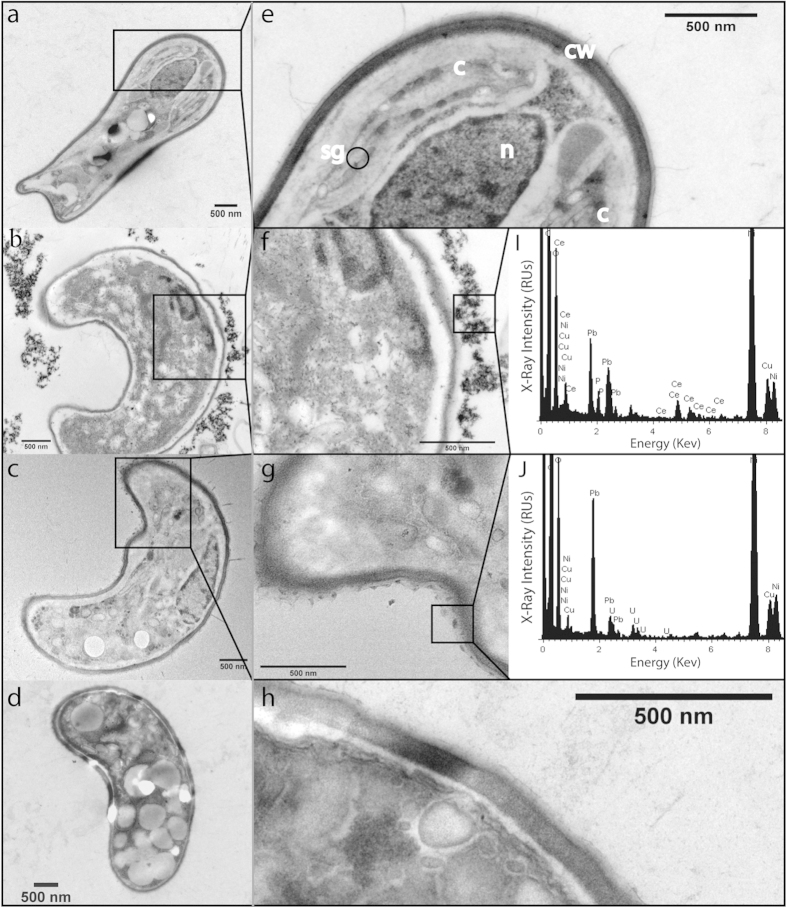
Selected Transmission electron microscopy (TEM) micrographs of *P. subcapitata* non-exposed (a,e) and exposed to CNP1 (b,f), CNP2 (c,g) and CNP1-PO_4_^−3^ (d,h) for 72 h, accompanied with XEDS spectrum (i, j). N = nucleus; C = chloroplast; SG = starch grain; CW = cell wall.

Attachment to the algal envelope was further confirmed by TEM-XEDS. As shown in Fig. 7(b,f), CNP1 nanoparticles clearly attached to the outer cell wall as several layers of electron-dense particles surrounding the cell wall were found. No evidence of internalization was observed. XEDS analysis assigned Ce as the main constituent of attached particles. The electron-dense nanoparticles were absent in non-exposed cells (a, e) or cells exposed to CNP2 (c, g). XEDS analysis already confirmed the absence of Ce around the cell wall. Treatment with phosphate (d, h) prevented nanoparticle attachment and no Ce was detected around the cell wall either. It is worth noticing that CNP1 (clearly seen in Fig. 7f) induced cell wall detachment from the cytoplasmic membrane, cell shrinking and disorganization as internal structures such as the nucleus, chloroplast or storage bodies could not be distinguished, indicating cell damage. CNP2 or CNP1-phosphate did not induce significant ultrastructural cell changes.

**Figure 8:**
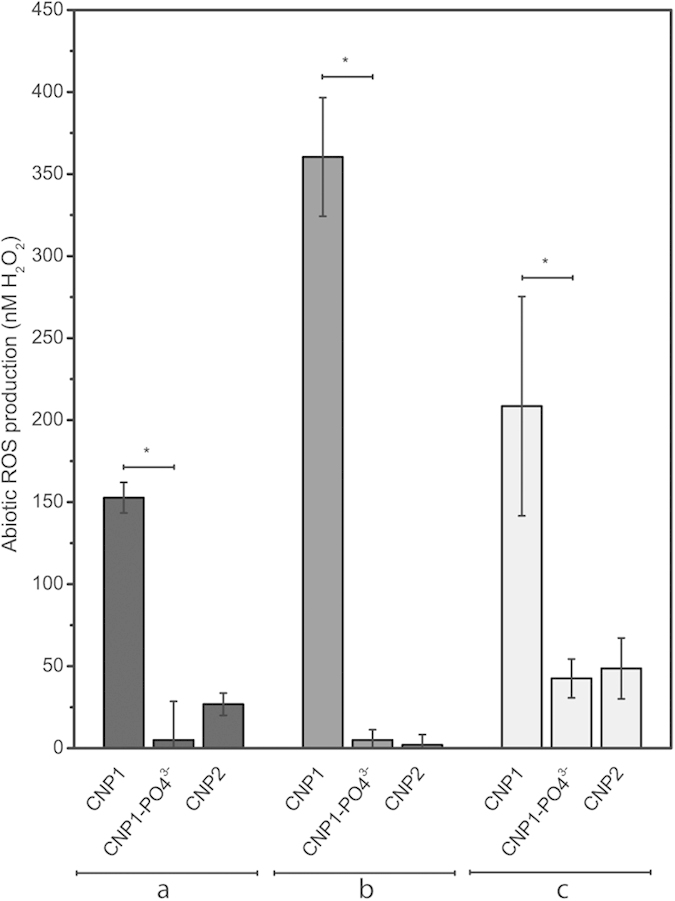
ROS production by 10 mgL^−1^ CNPs in ddH_2_O (a), in OECD-medium (b) and OECD-medium after 24h exposure to *P. subcapitata* to CNP1, CNP2 and CNP1-PO_4_^3−^ (c) assessed by OxiSelect™ *in vitro* ROS/RNS Assay Kit. ROS/RNS was expressed as H_2_O_2_ equivalent concentration.

In the present study, we have found that CNP1 induced ROS formation in the algal cells (Fig. 5) which, in the end, may result in oxidative stress. Interestingly, we have also found that treatment with phosphate prevented ROS formation. The relevant question is how adsorbed nanoceria induces ROS formation. Thill *et al.*^22^ and Zeyons *et al.*^23^ have found a reduction of surface Ce^4+^ to Ce^3+^ when particles are tightly adsorbed to *Escherichia coli* cell walls and have suggested that oxidative stress was triggered by the oxidant activity of Ce^4+^. In our study, we found that toxicity depended on % surface Ce^3+^, so that the reactivity of Ce^3+^ sites and not Ce^4+^ is the most likely source of ROS in the algal cells. Xia *et al.*^39^ found that nanoceria induced hydrogen peroxide production abiotically. Based on these findings, we evaluated the possibility of spontaneous abiotic ROS generation by CNP1, CNP2 and CNP1 treated with phosphate by using the OxiSelect™ *in vitro* ROS/RNS Assay Kit. As shown in Fig. 8, both in distilled water (a) and OECD algal culture medium (b), CNP1 induced significant production of ROS/RNS (152.7 nM as H_2_O_2_ equivalent concentration in H_2_O and almost twofold in OECD medium), while CNP2 or CNP1 treated with phosphate did not. In the experiment depicted in Fig. 8c, algal cells were exposed 24 h to the CNPs, then removed by centrifugation and ROS/RNS production was assayed in the supernatant. CNP1 significantly induced ROS/RNS formation (208.5 nM) while CNP2 and CNP1-phosphate did not induce significant ROS formation.

## Discussion

There are contradictory reports on whether nanoceria may act as an oxidant causing toxicity or as an antioxidant being able to scavenge free radical, and protect the cells from oxidative damage. A variety of different nanoceria particles have been used in the bioassays. The tested particles have been synthesized by a variety of methods and have been tested in many different cell types and organisms. In some of the organisms, internalization of nanoceria particles has been observed while not in others. Different mechanisms of nanoceria toxicity have been proposed, although in most cases, toxicity seemed to be related to oxidative stress. Thus, there is a disparity of data on biological activity of nanoceria and no clear consensus on which nanoparticle properties/characteristics are responsible of the observed effects.

As found in this study and reported previously, nanoceria internalization usually does not occur in organisms such as bacteria and algae with thick cell walls^23^,^24^,^25^,^27^,^28^ with a recently reported exception using PVP coated nanoceria^40^. However, nanoceria is able to internalize in human and animal cell lines and tissues^10^,^14^,^41^. Independently of internalization, evidence for nanoceria toxicity has been found in many of the tested cell systems and organisms. For internalized nanoceria, toxicity has been found to be related to lysosomal injury^10^,^41^ and oxidative stress^14^,^21^. For non-internalized nanoceria toxicity seems to be mediated by direct contact of nanoceria to cell walls of algae and bacteria^22^,^23^,^26^,^28^. Several mechanisms have been postulated to explain how non-internalized nanoceria may exert toxicity: interference with the nutrient transport functions of the membrane^23^, mechanical damage membrane disruption^24^,^25^, or ROS generation and oxidative stress induction^22^,^23^,^26^. The observed abiotic production of ROS by CNP1, most probably hydrogen peroxide, is in agreement with Xia *et al.*^39^ and Zhao *et al.*^42^ observations. Hydrogen peroxide is able to freely diffuse across cell walls and membranes. Heckert *et al.*^15^ reported that Ce^3+^ ions were capable of redox-cycling with hydrogen peroxide to generate ROS such as hydroxyl radicals. They suggested that surface Ce^3+^ sites rich in oxygen vacancies could be responsible of ROS production by nanoceria. The hydrogen peroxide abiotically produced by CNP1, through oxidative reactions, may generate damaging radicals such as hydroxyl radicals which cause cellular damage.

A step forward to start understanding the enigma of the biological activity of nanoceria are the studies by Ji *et al.*^43^ and Lin *et al.*^41^ in which a library of nanoceria rods with increasing aspect ratio (range between 1 to > 100) was constructed and toxicity tested in a human myeloid cell line and in two animal models, mouse lung and zebrafish gastrointestinal tract. They found that nanorods with an aspect ratio ≥ 22 induced cytotoxicity in the human cell line. They also found that the toxicological profile of the tested nanorods in both animal models depended on the aspect ratio, although this was clearly demonstrated only with the longest rod. However, these observations may only be relevant for internalized nanoceria.

In the present study, we demonstrate that neither shape, concentration, surface charge (ζ-potential), synthesis method nor nominal size had any influence in the observed toxic effects and present for the first time solid evidence of the involvement of % surface Ce^3+^ in toxicity of nanoceria in an environmentally relevant organism. Our results clearly showed that % surface Ce^3+^ correlated with toxicity and was the main driver explaining the observed toxic effect of nanoceria: CNP1 with the highest % surface Ce^3+^ (58%) was also the most toxic followed by CNP5 with a % surface Ce^3+^ of 40 whereas CNP2, CNP3 and CNP4 with lower % surface Ce^3+^ values (between 26–36%) were apparently safe for the model organism. The fact that a relatively small difference in % surface Ce^3+^ (40% for CNP5 which is toxic *vs.* 36% for CNP3 which is non- toxic) accounts for a larger biological effect may be explained by the differential catalytic activity of nanoparticles with varying Ce^3+^/Ce^4+^ ratios. Nanoceria with higher Ce^3+^ on the surface can efficiently scavenge superoxide radicals (superoxide dismutase mimetic activity) and produce H_2_O_2_ which becomes toxic to the cells. This is the case with CNP1 and CNP5 while CNP2, CNP3 and CNP4 are less active towards scavenging superoxide radicals^15^,^44^. In fact, nanoceria with lower Ce^3+^ and therefore higher Ce^4+^ on the surface (CNP2, CNP3 and CNP4) exhibit catalase mimetic activity^16^ which breaks down H_2_O_2_ to molecular oxygen, protecting the cells against this toxic reactive oxygen species (see below in the Discussion with phosphate treated CNP1). We hypothesize that in a narrow range of surface Ce^3+^ (between 40 and 30% surface Ce^3+^), there seems to be a shift from superoxide dismutase activity to catalase mimetic activity but the mechanisms underneath and whether the observed biological effect reported here may also happen in other cellular systems need further investigation.

It was further demonstrated that surface Ce^3+^ was the source of toxicity as blocking the Ce^3+^ sites of CNP1 with phosphate prevented toxicity and even stimulated growth and slightly reduced ROS levels with respect to the control. This stimulation could be due to an increase of the catalase mimetic activity of phosphate treated nanoceria as found by Singh *et al.*^18^.

Colloidal destabilization by Fe treatment only significantly increased toxicity of the already toxic CNP1. As nanoceria was not internalized, it was proposed that abiotically generated ROS, probably hydrogen peroxide, was the inducing factor of the observed oxidative stress.

Systematic studies such as those of Ji *et al.*^43^ and Lin *et al.*^41^ and those presented here might be useful to untangle the main drivers of the biological activity of synthesized nanoceria in different cell/organism systems and to define global descriptors of engineered nanoparticles (ENPs) bioactivity which may be useful in safer-by-design strategies and in Environmental Health and Safety (EHS) assessment of nanomaterials.

We demonstrate that the main driver of toxicity of cerium oxide nanoparticles is the percentage of Ce^3+^ at the surface of the nanoparticles: only the nanoparticles with the highest values exert toxicity in the ecologically relevant aquatic organism. As opposed to human and animal cell lines where nanoceria internalization usually occurs, nanoceria did not internalize in the alga. The mechanisms of toxicity rely primarily on the formation of abiotic ROS and on attachment of the nanoparticles to the cell wall which affect cell viability and ultrastructure. Blocking the Ce^3+^ sites of the toxic nanoparticles with phosphate prevented toxicity and even stimulated growth and slightly reduced ROS levels with respect to the control. This study gives clues towards a safer design of nanoparticles which may eventually end up in the aquatic environment.

## Methods

### Synthesis of Different Cerium Oxide Nanoparticles

In this study, five Cerium Oxide Nanoparticles (CNPs) were synthesized using different methods with varying % surface Ce^3+^, size and morphology. 99.999% pure cerium nitrate hexahydrate was used for all the preparation. CNP1 and CNP2 were synthesized using the wet chemical method, with H_2_O_2_ (CNP1) and NH_4_OH (CNP2) as oxidizing agents^45^. In order to prove that there was no H_2_O_2_ in CNP1 after the synthesis process, the Amplex® Red Hydrogen Peroxide/Peroxidase Assay Kit was performed following the manufacturer instructions (Life Technologies). Briefly, reactions containing 50 μM Amplex® Red reagent, 0.1 U/mL horseradish peroxidase (HRP) and increasing concentration of CNP1 (1, 10, 100 mg/l) were incubated for 30 minutes at room temperature. Fluorescence was then measured with a Fluorostar Omega plate reader (BMG LABTECH GmbH, Germany) using excitation at 530 nm and fluorescence detection at 590 nm. H_2_O_2_ was not detected in any of the concentration tested (see Supplementary Figure S3). No differences were found between CNP1 and control (with H_2_O). CNP3, CNP4 and CNP5 were prepared using the hydrothermal method, as described elsewhere^44^. The % surface Ce^3+^ for all the nanoparticles were tested several times over the experimental period in the aqueous environment (data not shown) and the % surface Ce^3+^/Ce^4+^ was stable for all these nanoparticles.

### CNPs chemistry

High Resolution Transmission Microscopy (HRTEM), FEI Tecnai F30 was used to analyze size and morphology of the particles. Selected Area Electron Diffraction patterns (SAED) of nanoparticles were analyzed to determine the crystallinity. Surface chemistry (Ce^3+^/Ce^4+^) ratios on the surface of nanoparticles was analyzed using X-Ray photoelectron spectroscopy as described elsewhere^46^. The optical properties were analyzed using Ultraviolet–Visible Spectrophotometer (Perkin Elmer, Lambda 750 S, 60 mm Int. Sphere). The dissolved fraction of CNPs was examined by centrifugal ultrafiltration (Sartorius AG, Goettingen, Germany) through a membrane with a nominal molecular weight limit of 50 kDa (Vivaspin 6). Suspensions were centrifuged for 15 min at 4000 rpm (Allegra X-12 Series, Beckman Coulter). Dissolution of CNPs was tested at a concentration of 10 mg/L in OECD media (algal growth medium; composition in Supplementary Table S4) under agitation (135 rpm) during 72 h. Nanoparticle suspensions were maintained under identical experimental conditions as the bioassays. The concentration of Ce^3+^ in the filtrate was related to the total Ce concentration as determined by ICP-MS. Cerium (III) chloride (CAS no. 7790–86–5) > 99.99% was purchased from Sigma-Aldrich. Water suspensions were prepared with high-purity water obtained from a Milipore Mili-Q system with a resistivity of at least 18 MΩ at 25 °C.

Hydrodynamic diameter and ζ-potential of the CNP suspensions in the different assay conditions were measured by Dynamic light scattering (DLS) and electrophoretic light scattering respectively using a Zetasizer Nano ZS particle size analyzer from Malvern Instruments Ltd. Measurements were essentially as described elsewhere^32^.

### Modification of surface chemistry and colloidal stability of CNPs in OECD media

0.1 mM [Fe_2_(SO_4_)_3_] was used to induce particle colloidal destabilization of CNPs suspension and heteroaggregation of CNP with algae^34^. Regarding surface chemistry properties, the phosphate treatment consisted on incubation (24 h) with equimolar concentration (100 μM) of phosphate buffer (13.8 g/L monosodium phosphate and 14.1 g/L disodium phosphate, pH 7.4) and CNPs. PO_4_^3−^ blocks the redox cycling between Ce^3+^ and Ce^4+^, which is essential for the catalytic activity of CNPs^18^,^37^. All the chemicals were from Merck (Darmstadt, Germany). The chosen concentrations of both buffers did not have any statistically significant effects on the algal studied parameters.

### Biological end-points

#### Growth inhibition

Growth inhibition of the green microalga *P. subcapitata* (Microbiotests. Inc.; Denmark) was performed essentially as described in Gonzalo, et al.^32^ following the standard TG 201^47^ guideline. Cells were routinely grown in 250 ml flasks on a rotatory shaker at 135 rpm in OECD standard culture medium (pH 8.2); the pH was regularly checked and remained unchanged during the experiment. Exposure experiments to CNPs suspensions were carried out in 1.8 mL of OECD culture medium in 24 well-plates. Growth inhibition experiments were performed during 72 h in the same experimental conditions at least in triplicate with serial dilutions. *In-vivo* fluorescence of chlorophyll (485 nm/645 nm excitation/emission) was measured daily as biomass surrogate as described elsewhere^32^ on a Synergy HTmulti-mode microplate reader (BioTek,USA).

#### Intracellular ROS

Intracellular ROS produced by *P. subcapitata* was measured by using the cell permeable fluorescent indicator 2′,7′-dichlorodihydrofluorescein diacetate (H_2_DCFDA, Invitrogen Molecular Probes; Eugene, OR, USA) as previously described^25^. 3% H2O2 (v/v) was used as a positive control for ROS formation. Fluorescence (488 and 530 nm) was monitored on a Synergy HT multi-mode microplate reader (BioTek, USA). Results were normalized for possible differences in cell number by the measured *in-vivo* fluorescence of chlorophyll of the sample.

#### Flow cytometric analyses

Chlorophyll (cell autofluorescence) and heteroaggregation (as FS *vs* SS distributions) of algal populations were evaluated using a Cytomix FL500 MPL flow cytometer equipped with an argon-ion excitation wavelength (488 nm), detector of forward (FS) and side (SS) light scatter and four fluorescence detectors (FL1:525, FL2:575, FL3:620 and FL4: 675 ± 20 nm) (Beckman Coulter Inc., Fullerton, CA, USA), as described previously^26^. The flow rate was set at 1 μLs^−1^ and at least 10000 events (algal cells) were counted. Non-algal particles were excluded from the analysis by setting an acquisition threshold value 1 for the forward scatter (FS) parameter. Chlorophyll red autofluorescence was collected with a 610 nm long band pass filter (FL4). Data acquisition was performed using MXP-2.2 software, and the analyses were performed using CXP-2.2 and Flowing Software 2.5.1 software. Fluorescence was analyzed in Log mode.

#### CNP-algal interaction by TEM and FT-IR

For transmission electron microscopy (TEM) analysis, algal cell suspensions exposed to the different CNP treatments were collected by centrifugation at low relative centrifugal forces (RCFs = 1500 g) during 3 min in order to reduce the chance for artifacts, cells where prepared essentially as previously described^32^. Cells were fixed in agar blocks in 3.1% glutaraldehyde in phosphate buffer (pH 7.2) for 3 h at 4 °C. Post-fixation was in osmium tetroxide in phosphate buffer for 2 h at 4 °C. Samples were dehydrated in ethanol and embedded in Durcupan resin (Fluka). Sample were sectioned in a Leica Reichert Ultracut S ultramicrotome, stained with uranyl acetate 2%. Ultrathin sections were visualized on a JEOL (JEM 1010) electron microscope (100 kV) or on a JEOL JEM 2100 (200 kV) coupled with XEDS (X-Ray Energy Dispersive Spectroscopy). All reagents used for TEM preparations were Electron Microscopy grade. For Fourier Transformed Infrared (FTIR) analyses, algal cell suspensions exposed to different CNPS treatment were centrifuged and 5 μL of pelletized cells were transferred to KBr dish and were dried for 2 h at 25 °C. Infrared spectra of the algal cell were obtained using a Bruker model IFs 66VFourier Transform Infrared spectrometer in transmission mode.

#### *In vitro* ROS assessment

Spontaneous ROS generation by CNPs was determined by using the OxiSelect *in vitro* ROS/RNS assay kit (Cell BioLabs, San Diego, USA). The kit was used according to the recommendations of the manufacturer. ROS and the reactive nitrogen species (RNS) content were determined in ddH_2_O and OECD algal exposure medium by fluorescence (480 nm/530 nm), measured in 96 well opake microttiter plates in a Synergy HT multi-mode microplate reader (BioTek, USA). Quantitative determinations of ROS/RNS content were estimated using a hydrogen peroxide standard curve. For the assay, 10 mg/L of CNPs suspensions were added to ddH_2_O or algal medium and ROS/RNS content was determined after 15 min. In addition, ROS/RNS content was also determined in used culture medium (medium where the cells were previously exposed to CNPs particles) after removing cells by centrifugation, 5 min, 10000 rpm.

### Statistical Analysis

Statistical analyses were performed by using R software 3.0.2. (The R Foundation for Statistical Computing©) and Rcmdr 2.0–4 package^48^. A one way ANOVA coupled with Tukey’s HSD (honestly significant difference) post-hoc test was performed for comparison of means. Statistically significant differences were considered to exist when *p* *<* *0.05*.

## Additional Information

**How to cite this article**: Pulido-Reyes, G. *et al.* Untangling the biological effects of cerium oxide nanoparticles: the role of surface valence states. *Sci. Rep.* **5**, 15613; 10.1038/srep15613 (2015).

## Supplementary Material

Supplementary Information

## Figures and Tables

**Table 1:** Physicochemical properties of the five tested Cerium Oxide Nanoparticles (CNPs). See Fig. 1 for more details.

Sample Name	% Ce^3+^	Morphology	Size from HRTEM (nm)	ddH_2_O	OECD medium		
				ζ-potential (mV)	ζ-potential (mV)	Effective Diameter (nm)	PDI*
CNP1	58	spheres	≈5	18.1 ± 0.7	−12.2 ± 0.64	793.8	0.455
CNP2	28	spheres	≈7	30.2 ± 1.5	−25.6 ± 2.54	298.5	0.659
CNP3	36	rod	>50	22.8 ± 0.72	−21.2 ± 0.78	511.6	0.809
CNP4	26	cube	≈50	−28.7 ± 1.25	−24.4 ± 1.03	256.1	0.503
CNP5	40	spheres	≈18	28.3 ± 0.59	−21.1 ± 1.01	286.9	0.589

^*^PDI = Polydispersity index.

## References

[b1] Hayes S. A., Yu P., O’Keefe T. J., O’Keefe M. J. & Stoffer J. O. The Phase Stability of Cerium Species in Aqueous Systems: I. E-pH Diagram for the Ce HClO 4 H 2 O System. Journal of The Electrochemical Society 149, C623–C630 (2002).

[b2] Reed K. *et al.* Exploring the properties and applications of nanoceria: is there still plenty of room at the bottom? Environmental Science: Nano 1, 390–405, 10.1039/C4EN00079J (2014).

[b3] Pang X., Li D. & Peng A. Application of rare-earth elements in the agriculture of china and its environmental behavior in soil. J Soils Sediments 1, 124–129, 10.1007/BF02987718 (2001). 12008295

[b4] Patil S., Kuiry S. C., Seal S. & Vanfleet R. Synthesis of Nanocrystalline Ceria Particles for High Temperature Oxidation Resistant Coating. Journal of Nanoparticle Research 4, 433–438, 10.1023/A:1021696107498 (2002).

[b5] Jasinski P., Suzuki T. & Anderson H. U. Nanocrystalline undoped ceria oxygen sensor. Sensors and Actuators B: Chemical 95, 73–77, 10.1016/S0925-4005(03)00407-6 (2003).

[b6] Croy J. R. *et al.* Support dependence of MeOH decomposition over size-selected Pt nanoparticles. Catalysis Letters 119, 209–216 (2007).

[b7] Giraldo J. P. *et al.* Plant nanobionics approach to augment photosynthesis and biochemical sensing. Nature materials 13, 400–408, 10.1038/nmat3890 (2014). 24633343

[b8] Das M. *et al.* Auto-catalytic ceria nanoparticles offer neuroprotection to adult rat spinal cord neurons. Biomaterials 28, 1918–1925 (2007). 17222903 10.1016/j.biomaterials.2006.11.036 PMC1913191

[b9] Tarnuzzer R. W., Colon J., Patil S. & Seal S. Vacancy engineered ceria nanostructures for protection from radiation-induced cellular damage. Nano letters 5, 2573–2577, 10.1021/nl052024f (2005). 16351218

[b10] Asati A., Santra S., Kaittanis C. & Perez J. M. Surface-charge-dependent cell localization and cytotoxicity of cerium oxide nanoparticles. ACS nano 4, 5321–5331 (2010). 20690607 10.1021/nn100816s PMC2947560

[b11] Karakoti A. S. *et al.* Nanoceria as Antioxidant: Synthesis and Biomedical Applications. JOM (Warrendale, Pa. : 1989) 60, 33–37, 10.1007/s11837-008-0029-8 (2008). PMC2898180 20617106

[b12] Campbell C. T. & Peden C. H. F. Oxygen Vacancies and Catalysis on Ceria Surfaces. Science 309, 713–714, 10.1126/science.1113955 (2005). 16051777

[b13] Esch F. *et al.* Electron Localization Determines Defect Formation on Ceria Substrates. Science 309, 752–755, 10.1126/science.1111568 (2005). 16051791

[b14] Zhang H. *et al.* Nano-CeO2 exhibits adverse effects at environmental relevant concentrations. Environmental science & technology 45, 3725–3730, 10.1021/es103309n (2011). 21428445

[b15] Heckert E. G., Karakoti A. S., Seal S. & Self W. T. The role of cerium redox state in the SOD mimetic activity of nanoceria. Biomaterials 29, 2705–2709, 10.1016/j.biomaterials.2008.03.014 (2008). 18395249 PMC2396488

[b16] Pirmohamed T. *et al.* Nanoceria exhibit redox state-dependent catalase mimetic activity. Chemical communications 46, 2736–2738, 10.1039/b922024k (2010). 20369166 PMC3038687

[b17] Dowding J. M., Dosani T., Kumar A., Seal S. & Self W. T. Cerium oxide nanoparticles scavenge nitric oxide radical (NO). Chemical communications 48, 4896–4898, 10.1039/c2cc30485f (2012). 22498787

[b18] Singh S. *et al.* A phosphate-dependent shift in redox state of cerium oxide nanoparticles and its effects on catalytic properties. Biomaterials 32, 6745–6753, 10.1016/j.biomaterials.2011.05.073 (2011). 21704369 PMC3143296

[b19] Naganuma T. & Traversa E. The effect of cerium valence states at cerium oxide nanoparticle surfaces on cell proliferation. Biomaterials 35, 4441–4453, 10.1016/j.biomaterials.2014.01.074 (2014). 24612920

[b20] Asati A., Santra S., Kaittanis C., Nath S. & Perez J. M. Oxidase-like activity of polymer-coated cerium oxide nanoparticles. Angewandte Chemie 48, 2308–2312, 10.1002/anie.200805279 (2009). 19130532 PMC2923475

[b21] Lin W., Huang Y. W., Zhou X. D. & Ma Y. Toxicity of cerium oxide nanoparticles in human lung cancer cells. International journal of toxicology 25, 451–457, 10.1080/10915810600959543 (2006). 17132603

[b22] Thill A. *et al.* Cytotoxicity of CeO2 Nanoparticles for Escherichia coli. Physico-Chemical Insight of the Cytotoxicity Mechanism. Environmental science & technology 40, 6151–6156, 10.1021/es060999b (2006). 17051814

[b23] Zeyons O. *et al.* Direct and indirect CeO2nanoparticles toxicity forEscherichia coliandSynechocystis. Nanotoxicology 3, 284–295, 10.3109/17435390903305260 (2009).

[b24] Rogers N. J. *et al.* Physico-chemical behaviour and algal toxicity of nanoparticulate CeO2 in freshwater. Environmental Chemistry 7, 50–60, 10.1071/EN09123 (2010).

[b25] Rodea-Palomares I. *et al.* Physicochemical characterization and ecotoxicological assessment of CeO2 nanoparticles using two aquatic microorganisms. Toxicological sciences: an official journal of the Society of Toxicology 119, 135–145, 10.1093/toxsci/kfq311 (2011). 20929986

[b26] Rodea-Palomares I. *et al.* An insight into the mechanisms of nanoceria toxicity in aquatic photosynthetic organisms. Aquatic toxicology 122-123, 133–143, 10.1016/j.aquatox.2012.06.005 (2012). 22797055

[b27] Van Hoecke K. *et al.* Fate and effects of CeO2 nanoparticles in aquatic ecotoxicity tests. Environmental science & technology 43, 4537–4546 (2009). 19603674 10.1021/es9002444

[b28] Manier N., Bado-Nilles A., Delalain P., Aguerre-Chariol O. & Pandard P. Ecotoxicity of non-aged and aged CeO2 nanomaterials towards freshwater microalgae. Environmental pollution 180, 63–70, 10.1016/j.envpol.2013.04.040 (2013). 23727569

[b29] Rohder L. A., Brandt T., Sigg L. & Behra R. Influence of agglomeration of cerium oxide nanoparticles and speciation of cerium(III) on short term effects to the green algae Chlamydomonas reinhardtii. Aquatic toxicology 152, 121–130, 10.1016/j.aquatox.2014.03.027 (2014). 24747084

[b30] Kuchibhatla S. V. *et al.* Influence of Aging and Environment on Nanoparticle Chemistry—Implication to Confinement Effects in Nanoceria. The journal of physical chemistry. C, Nanomaterials and interfaces 116, 14108–14114, 10.1021/jp300725s (2012). 23573300 PMC3618908

[b31] McCormack R. N. *et al.* Inhibition of Nanoceria’s Catalytic Activity due to Ce3+Site-Specific Interaction with Phosphate Ions. The Journal of Physical Chemistry C 118, 18992–19006, 10.1021/jp500791j (2014).

[b32] Gonzalo S. *et al.* A colloidal singularity reveals the crucial role of colloidal stability for nanomaterials *in-vitro* toxicity testing: nZVI-microalgae colloidal system as a case study. PloS one 9, e109645, 10.1371/journal.pone.0109645 (2014). 25340509 PMC4207682

[b33] Booth A. *et al.* Freshwater dispersion stability of PAA-stabilised cerium oxide nanoparticles and toxicity towards Pseudokirchneriella subcapitata. The Science of the total environment 505, 596–605, 10.1016/j.scitotenv.2014.10.010 (2015). 25461062

[b34] Gregory J. Particles in water: properties and processes. (CRC Press, 2005).

[b35] Stankus D. P., Lohse S. E., Hutchison J. E. & Nason J. A. Interactions between Natural Organic Matter and Gold Nanoparticles Stabilized with Different Organic Capping Agents. Environmental science & technology 45, 3238–3244, 10.1021/es102603p (2011). 21162562

[b36] Dowding J. M. *et al.* Cellular interaction and toxicity depend on physicochemical properties and surface modification of redox-active nanomaterials. ACS nano 7, 4855–4868, 10.1021/nn305872d (2013). 23668322 PMC3700371

[b37] Xue Y. *et al.* The vital role of buffer anions in the antioxidant activity of CeO2 nanoparticles. Chemistry 18, 11115–11122, 10.1002/chem.201200983 (2012). 22807390

[b38] Jalilpour M. & Fathalilou M. Effect of aging time and calcination temperature on the cerium oxide nanoparticles synthesis via reverse co-precipitation method. International Journal of the Physical Sciences 7 (2012).

[b39] Xia T. *et al.* Comparison of the mechanism of toxicity of zinc oxide and cerium oxide nanoparticles based on dissolution and oxidative stress properties. ACS nano 2, 2121–2134 (2008). 19206459 10.1021/nn800511k PMC3959800

[b40] Taylor N. S. *et al.* Molecular toxicity of cerium oxide nanoparticles to the freshwater alga Chlamydomonas reinhardtii is associated with supra-environmental exposure concentrations. Nanotoxicology 1–10, 10.3109/17435390.2014.1002868 (2015). PMC4819577 25740379

[b41] Lin S. *et al.* Aspect Ratio Plays a Role in the Hazard Potential of CeO2 Nanoparticles in Mouse Lung and Zebrafish Gastrointestinal Tract. ACS nano 8, 4450–4464, 10.1021/nn5012754 (2014). 24720650 PMC4059546

[b42] Zhao L. *et al.* Stress Response and Tolerance of Zea mays to CeO2 Nanoparticles: Cross Talk among H2O2, Heat Shock Protein, and Lipid Peroxidation. ACS nano 6, 9615–9622, 10.1021/nn302975u (2012). 23050848 PMC4326050

[b43] Ji Z. *et al.* Designed Synthesis of CeO2 Nanorods and Nanowires for Studying Toxicological Effects of High Aspect Ratio Nanomaterials. ACS nano 6, 5366–5380, 10.1021/nn3012114 (2012). 22564147 PMC3651271

[b44] Sakthivel T. *et al.* Morphological phase diagram of biocatalytically active ceria nanostructures as a function of processing variables and their properties. Chem Plus Chem 78, 1446–1455 (2013). 10.1002/cplu.201300302 31986658

[b45] Das S. *et al.* The induction of angiogenesis by cerium oxide nanoparticles through the modulation of oxygen in intracellular environments. Biomaterials 33, 7746–7755, 10.1016/j.biomaterials.2012.07.019 (2012). 22858004 PMC4590782

[b46] Deshpande S., Patil S., Kuchibhatla S. V. & Seal S. Size dependency variation in lattice parameter and valency states in nanocrystalline cerium oxide. Applied Physics Letters 87, 133113-133113-133113 (2005).

[b47] OECD. Test No. 201: Freshwater Alga and Cyanobacteria, Growth Inhibition Test.

[b48] Fox J. Getting started with the R commander: a basic-statistics graphical user interface to R. Journal of statistical software 14, 1–42 (2005).

