# Supplementary material for: Untangling the biological effects of cerium oxide nanoparticles: the role of surface valence states
Source: Sci Rep. 2015 Oct 22;5:15613. doi: 10.1038/srep15613 (PMC4615008; doi:10.1038/srep15613)
Supplement: Supplementary Information [file srep15613-s1.pdf]

Untangling the biological effects of cerium oxide nanoparticles: the role of surface valence states

List of Authors

Author nº: 1
Name: Gerardo Pulido-Reyes
Institution: Departamento de Biología, Facultad de Ciencias, Universidad Autónoma de Madrid, E-28049, Spain
Email: gerardo.pulido@uam.es

Author nº: 2
Name: Ismael Rodea-Palomares
Institution: Departamento de Biología, Facultad de Ciencias, Universidad Autónoma de Madrid, E-28049, Spain
Email: ismael.rodea@uam.es

Author nº: 3
Name: Soumen Das
Institution: Advance Material Processing Analysis Center and Nanoscience Technology Center, University of Central Florida, Florida 32826, United States
Email: soumen.das@ucf.edu

Author nº: 4
Name: Tamil Selvan Sakthivel
Institution: Advance Material Processing Analysis Center and Nanoscience Technology Center, University of Central Florida, Florida 32826, United States
Email: tselvan06@gmail.com

Author nº: 5
Name: Francisco Leganes
Institution: Departamento de Biología, Facultad de Ciencias, Universidad Autónoma de Madrid, E-28049, Spain
Email: francisco.leganes@uam.es

Author nº: 6
Name: Roberto Rosal
Institution: Departamento de Ingeniería Química, Universidad de Alcalá, E-28871, Alcalá de Henares, Madrid, Spain
Email: roberto.rosal@uah.es

Author nº: 7
Name: Sudipta Seal
Institution: Advance Material Processing Analysis Center and Nanoscience Technology Center, University of Central Florida, Florida 32826, United States // Material Science and Engineering, Collage of Medicine, University of Central Florida, Florida 32826, USA
Email: sudipta.seal@ucf.edu

Author nº: 8 (Submitting Author)
Name: Francisca Fernández-Piñas
Institution: Departamento de Biología, Facultad de Ciencias, Universidad Autónoma de Madrid, E-28049, Spain
Email: francisca.pina@uam.es

Suplementary Table S1.- Spontaneous cerium dissolution in the exposure media for all CNPs was tested by performing ICP-MS analyses of ultrafiltrated samples (10 mg/l).

Sample	Concentration (mg/l)	Stand. Dev
CNP1	0,00045	4.13%
CNP2	0,00001	29.22%
CNP3	0,00081	8.95%
CNP4	0,00003	4.05%
CNP5	n.d.*	-
CNP6	n.d.*	-
CNP7	n.d.*	-

*No detected

Supplementary Figure S1.- Effect of free ion Ce^{3+} on growth of *P. subcapitata*.

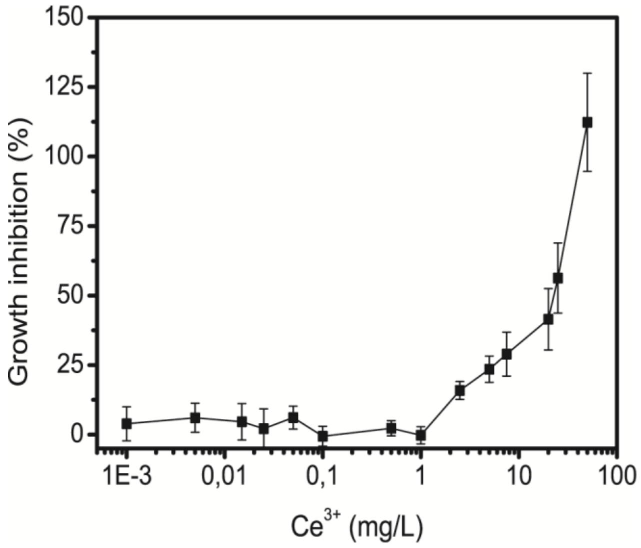

Supplementary Table S2.- Summary table of correlation analyses between algal growth inhibition and different tested factors, showing the adjusted R^2 values and their associated *p*-values (ANOVA, F<P). $\alpha = 0.05$ is stablished as significance criterion (marked by asterisks).

**Tested factors**	R^2	***p*-value**
Surface Ce^{3+}	0.706	0.047*
ζ-Potential	0.693	0.049*
Nominal Size	0.007	0.384
Effective Size	0.152	0.281

Supplementary Figure S2. Box plots which show agal growth inhibition as function of nanoparticle shape (cube: CNP4, rod: CNP3 and sphere: CNP1, CNP2 and CNP5; Figure S2a) and just as function of spheric-shaped nanoceria (CNP1, CNP2 and CNP5; Figure S2b). A one way ANOVA coupled with Tukey's HSD (honestly significant difference) post-hoc test was performed for comparison of means. Statistically significant differences (α = *0.05*) are marked by asterisks.

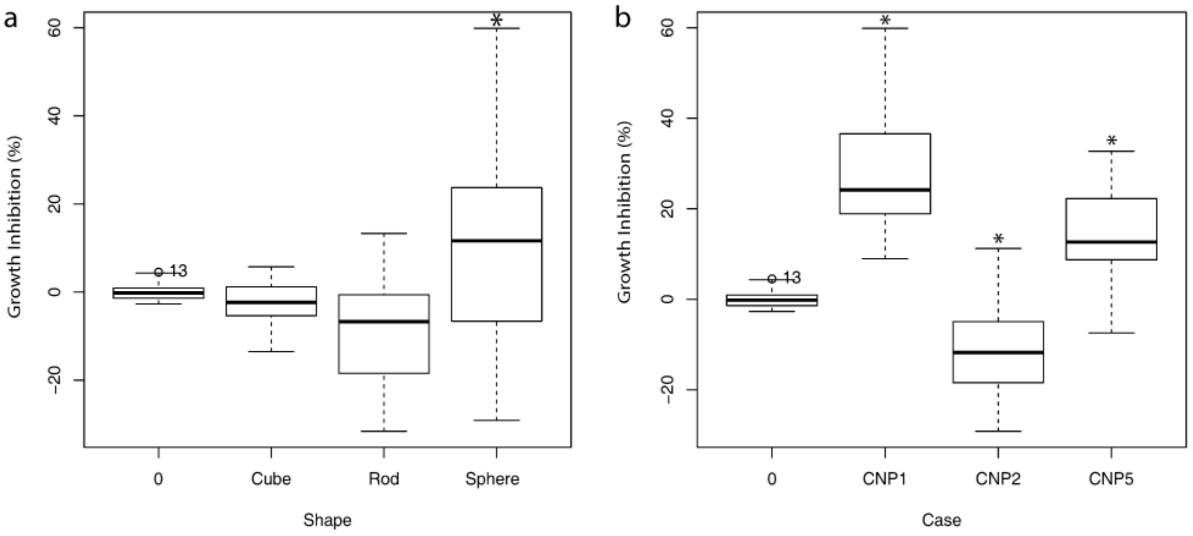

Suplementary Table S3.- ζ-potential and effective diameter of CNP1 and CNP2 with the addition of Fe or phosphate.

10 mgL^{-1} OECD medium						
	**0.1 mM Fe**			**0.2 mM Phosphate**		
**Sample Name**	**ζ-potential (mV)**	**Effective Diameter (nm)**	**PDI***	**ζ-potential (mV)**	**Effective Diameter (nm)**	**PDI***
**CNP1**	10.15 ± 0.889	787.3	0.979	-19.90 ± 0.557	817.2	0.526
**CNP2**	-15.30 ± 1.9	1073	0.362	-10.14 ± 1.06	450.7	0.573

*PDI=Polydispersity index

Supplementary Figure S3.- Detection of H_2O_2 using the Amplex® Red Hydrogen Peroxide/Peroxidase Assay Kit. Reactions containing 50 µM Amplex® Red reagent, 0.1 U/mL HRP and the indicated amount of CNP1 (a) or H_2O_2 as standard curve (b) were incubated for 30 minutes at room temperature. The fluorescence (excitation 535 nm, emission 595 nm) was measured on a Fluorostar Omega plate reader (BMG LABTECH GmbH, Germany). Background fluorescence, determined for a control reaction (H_2O), has been subtracted from each value.

a

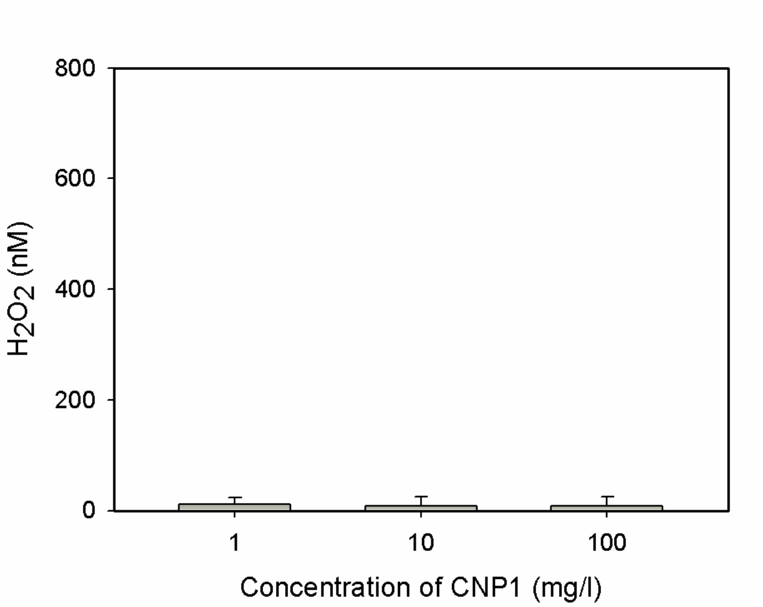

b

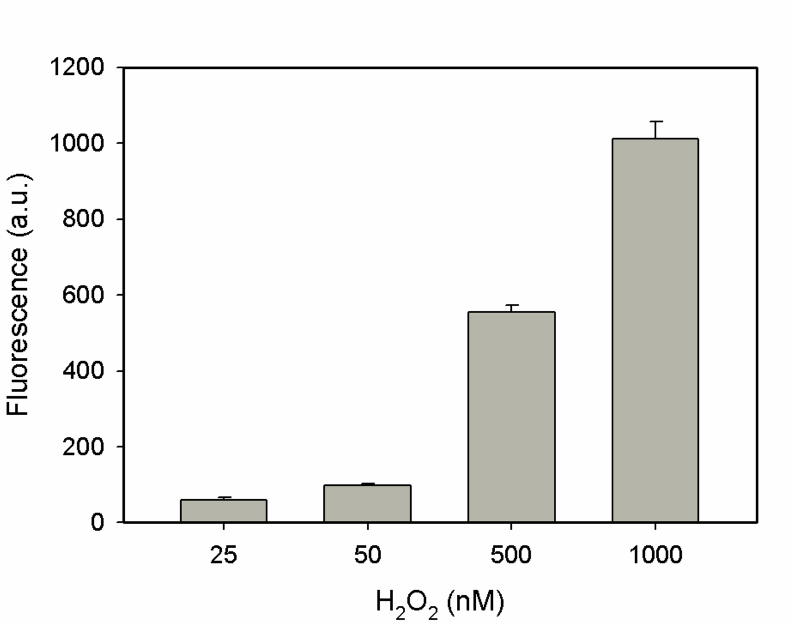

Supplementary Table S4.- Composition of the OECD TG 201 medium.

Component	OECD	
	mg/L	mM
$NaHCO_3$	50.0	0.595
$NaNO_3$<br>NH_4Cl	15.0	0.280
$MgCl_2 \cdot 6(H_2O)$	12.0	0.0590
$CaCl_2 \cdot 2(H_2O)$	18.0	0.122
$MgSO_4 \cdot 7(H_2O)$	15.0	0.0609
K_2HPO_4<br>KH_2PO_4	1.60	0.00919
$FeCl_3 \cdot 6(H_2O)$	0.0640	0.000237
$Na_2EDTA \cdot 2(H_2O)$	0.100	0.000269
H_3BO_3	0.185	0.00299
$MnCl_2 \cdot 4(H_2O)$	0.415	0.00210
$ZnCl_2$	0.00300	0.0000220
$CoCl_2 \cdot 6(H_2O)$	0.00150	0.00000630
$Na_2MoO_4 \cdot 2(H_2O)$	0.00700	0.0000289
$CuCl_2.2(H_2O)$	0.00001	0.00000006
